# The Optimal PEG for Kidney Preservation: A Preclinical Porcine Study

**DOI:** 10.3390/ijms19020454

**Published:** 2018-02-03

**Authors:** Sebastien Giraud, Raphael Thuillier, Ricardo Codas, Emily Manguy, Benoit Barrou, Alexandre Valagier, Alexis Puichaud, Lionel Badet, Emmanuelle Nicolas, Michel Eugene, Thierry Hauet

**Affiliations:** 1 Inserm, U1082, 86000 Poitiers, France; sebastien.giraud@chu-poitiers.fr (S.G.); Raphael.Thuillier@chu-poitiers.fr (R.T.); ricardo.codas-duarte@chu-lyon.fr (R.C.); emilie.manguy@gmail.com (E.M.); benoit.barrou@gmail.com (B.B.); Alexandre.Valagier@ch-niort.fr (A.V.); Alexis.Puichaud@ch-rochefort.fr (A.P.); lionel.badet@chu-lyon.fr (L.B.); michel.eugene@gmail.com (M.E.); 2 Faculté de Medecine et Pharmacie, Université de Poitiers, 86000 Poitiers, France; 3 CHU de Poitiers, Service de Biochimie, 86000 Poitiers, France; Emmanuelle.NICOLAS@chu-poitiers.fr; 4 FHU SUPORT, 86000 Poitiers, France; 5 Faculté de Medecine, Université Claude Bernard Lyon 1, 69622 Villeurbanne, France; 6 Réseau CENTAURE, 92160 Antony, France; 7 Service d’Urologie et Transplantation, Groupe Hospitalier Pitié Salpetriere, AP-HP, 75013 Paris, France; 8 Faculté de Medecine, Université Pierre et Marie Curie, Paris VI, 75005 Paris, France; 9 Plate-forme MOPICT-IBiSA, INRA, Unité de Transplantation Expérimentale, GEPA, Domaine du Magneraud, 17700 Surgères, France; 10 INSERM U1082, CHU de Poitiers, 2 rue de la Miletrie CS 90577, 86021 Poitiers, France

**Keywords:** graft preservation, ischemia reperfusion injury, kidney transplantation, PEG, polyethylene glycol

## Abstract

University of Wisconsin (UW) solution is not optimal for preservation of marginal organs. Polyethylene glycol (PEG) could improve protection. Similarly formulated solutions containing either 15 or 20 g/L PEG 20 kDa or 5, 15 and 30 g/L PEG 35 kDa were tested in vitro on kidney endothelial cells, ex vivo on preserved kidneys, and in vivo in a pig kidney autograft model. In vitro, all PEGs provided superior preservation than UW in terms of cell survival, adenosine triphosphate (ATP) production, and activation of survival pathways. Ex vivo, tissue injury was lower with PEG 20 kDa compared to UW or PEG 35 kDa. In vivo, function recovery was identical between UW and PEG 35 kDa groups, while PEG 20 kDa displayed swifter recovery. At three months, PEG 35 kDa 15 and 30 g/L animals had worse outcomes than UW, while 5 g/L PEG 35 kDa was similar. PEG 20 kDa was superior to both UW and PEG 35 kDa in terms of function and fibrosis development, with low activation of damage pathways. PEG 20 kDa at 15 g/L was superior to 20 g/L. While in vitro models did not discriminate between PEGs, in large animal models of transplantation we showed that PEG 20 kDa offers a higher level of protection than UW and that longer chains such as PEG 35 kDa must be used at low doses, such as found in Institut George Lopez (IGL1, 1g/L).

## 1. Introduction

Ischemia–reperfusion injury (IRI) is unavoidable in the majority of transplant situations. As it strongly correlates with delayed graft function [1] and chronic graft failure [2,3], the need for preservation solutions aimed at lowering IRI is paramount.

The University of Wisconsin solution (UW) is the current gold standard in static preservation. However, high potassium [4] induces cellular depolarization and vasoconstriction, impairing organ perfusion during washout and reperfusion [5], and hydroxyethyl starch (HES), a colloid used in UW, causes red blood cell aggregation [6], tubular damage, and macrophage invasion [7]. Several other solutions exist, such as Histidine-Tryptophan-Ketoglutarate or Celsior, but their superiority over UW is not confirmed [8,9]. There is thus a need for new solutions with superior preservation properties and reduced side effects. We demonstrated that using polyethylene glycol (PEG) in a plasma-like ionic balance solution provided significant superiority over UW for early function recovery, histological damage, and long term outcomes [10]. Other solutions such as Institut George Lopez (IGL1) and Polysol use PEG in their compositions, however reports of their efficacy are conflicting [8] or have a short follow up with no evaluation of long term outcome [11,12]. 

PEGs offer several advantages for preservation: they are non-toxic, neutral, water soluble, and their high affinity for water molecules along their chain creates layers of “structured water” when adsorbed to the cell surface. This prevents the creation of the immunological synapse [13], offering the possibility of “immunocamouflage” [14,15]. Protection from the immune system is also provided by PEG-induced change of cell surface potential gradient. These properties are entirely dependent on chain length [15]. However, the literature on the use of PEG in preservation is confusing, as two chain lengths (35 and 20 kDa) are used, and some reports highlight the benefits of one, with work performed with the other [16,17,18]. Hence, it is paramount to determine the best polymer size and concentration for optimal protection [19].

We demonstrated the superiority of SCOT^®^ (Solution de Conservation des Organes et des Tissus) solution over UW and IGL-1 in pig kidney low-mismatch allografts [20,21]. SCOT^®^ also showed superiority in islet preservation, islet yield, reduced graft immunogenicity, and improved graft survival in pancreatic islet transplantation [22]. This prompted us to base our test on the ionic composition of SCOT^®^ (Table S1), with varying concentrations and chain lengths of PEG. In terms of PEG types, SCOT^®^ includes 30 g/L of 20 kDa PEG, while IGL-1 uses 1 g/L 35kDa PEG, and we have previously determined that higher doses of PEG 20 kDa (50 g/L) are inferior to 30 g/L [10]. We thus endeavored to compare 20 and 35 kDa, with PEG 20 kDa tested at two lower dosages, 20 and 15 g/L; while PEG 35 kDa was tested at incremented doses compared to IGL, namely 5, 15, and 30 g/L. In terms of molarity, 20 g of PEG 20 kDa is close to 30 g of PEG 35 kDa (1 versus 0.9 mM respectively). This experimental design allows us to focus on the specific role of PEGs. 

All were compared to UW, the gold standard, in a pig model of kidney autotransplantation. This animal is of particular interest as its kidney is multilobular and multipapillar [23,24], with a complex network of blood vessels, also found in humans but not in mouse, rats or dogs. A 3-month follow-up allows for evaluation of both early and chronic outcome, without the bias of immunosuppression [25], as it permits for investigation of interstitial fibrosis and tubular atrophy (IFTA) development [26,27].

## 2. Results

### 2.1. Protection from Hypoxia in Endothelial Cells

**Figure 1:**
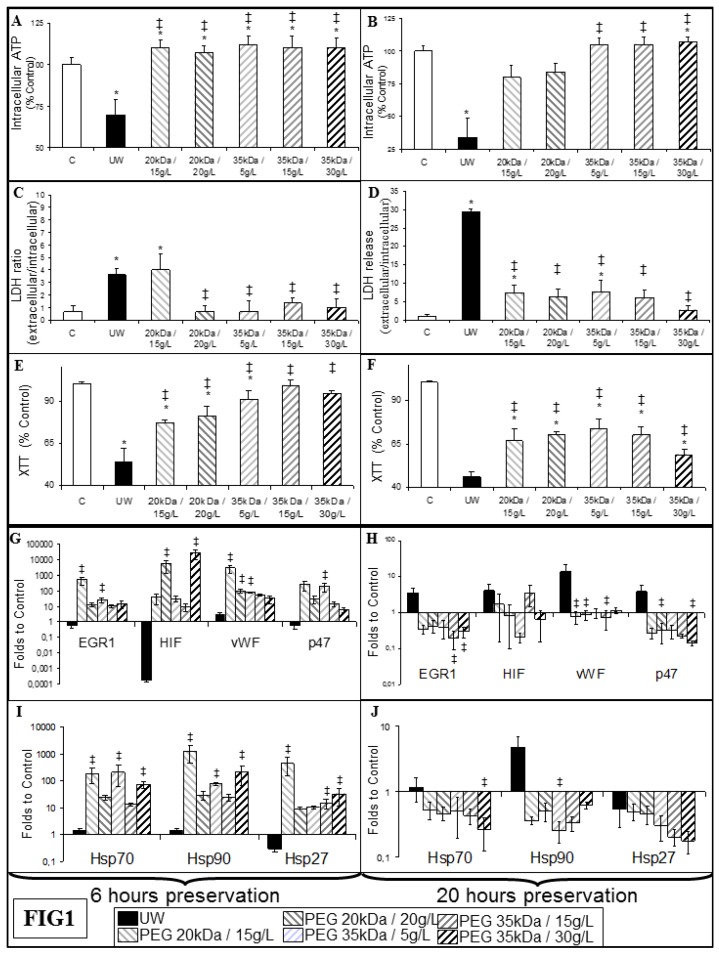
In vitro evaluation of endothelial cell resistance to ischemia–reperfusion injury (IRI). Primary porcine endothelial cells underwent 6 h (**A**,**C**,**E**,**G**,**I**) or 20 h (**B**,**D**,**F**,**H**,**J**) hypoxia in University of Wisconsin (UW) or polyethylene glycol (PEG) solutions. (**A**,**B**) Cellular adenosine triphosphate (ATP) content expressed as percentage of ATP in control cells (no hypoxia); (**C**,**D**) Cell death determined by calculating the ratio between extracellular over intracellular Lactate DesHydrogenase (LDH); (**E**,**F**) Mitochondrial Succinate Deshydrogenase activity assessment by 2,3-bis-(2-methoxy-4-nitro-5-sulfophenyl)-2*H*-tetrazolium-5-carboxanilide (XTT) assay, expressed as percentage of viability in Control cells (no hypoxia); (**G**,**H**,**I**,**J**): RTqPCR evaluation, mRNA expression is calculated as folds to control cells. Control are cells incubated in regular medium for 48 h. Shown are mean + SEM. Statistics: * *p* < 0.05 versus Control; ‡ *p* < 0.05 to UW. *n* = 3.

We measured the impact of hypoxia on endothelial cells. After both 6 and 24 h, cells preserved in UW showed significantly decreased ATP content (Figure 1A,B), increased release of Lactate DesHydrogenase (LDH, Figure 1C,D) and decreased Mitochondrial Succinate Dehydrogenase (complex II) activity (Figure 1E,F), a sign of cellular decay. All PEG conditions prevented a decrease in ATP content at both 6 and 20 h preservation (Figure 1A,D). After 6 h preservation, all PEG conditions, with the exception of PEG 20 kDa/15 g, were superior to UW in the LDH assay; after 20 h, the superiority of PEG solutions was confirmed over UW in this test (Figure 1C,D). A similar conclusion was reached when observing the results of the 2,3-bis-(2-methoxy-4-nitro-5-sulfophenyl)-2*H*-tetrazolium-5-carboxanilide (XTT) test.

RTqPCR on these cells after 6 h showed increased production of stress markers such as Early growth response factor 1 (EGR1), hypoxia inducible factor 1 alpha (HIF1α), von Willebrand factor (vWF), and NADPH oxidase subunit Phoxp47 (p47) (Figure 1G). We also observed high levels of expression for heat shock proteins (HSP) 27, 70, and 90 (Figure 1I). UW-preserved cells did not show such expression at this time, but after 20 h there was a trend towards expression of EGR1, HIF1α, vWF, p47, and Hsp90, while PEG preserved cells showed control or below control level of expression for all markers (Figure 1H,J).

### 2.2. Protection from Ischemia Ex Vivo

**Figure 2:**
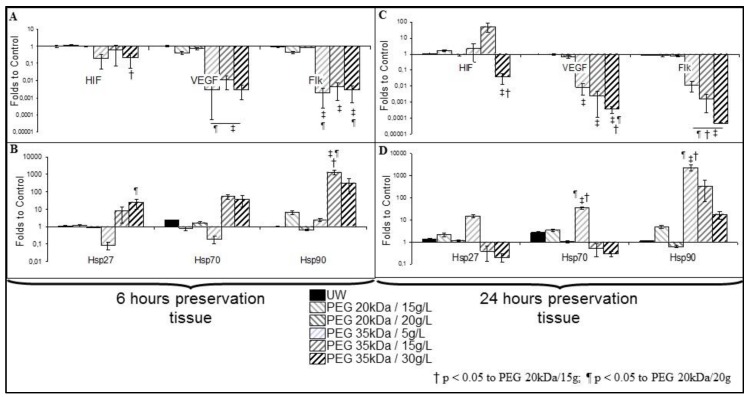
Reverse Transcription quantitative Polymerase Chain Reaction (RTqPCR) on preserved kidneys. Porcine kidneys underwent 6h (**A**,**B**) or 24h (**C**,**D**) preservation in UW or PEG solutions. Messenger RNA (mRNA) expression is calculated as fold over control cells, incubated in regular medium for 48 h. Shown are mean + SEM. Statistics: ‡ *p* < 0.05 to UW; † *p* < 0.05 to PEG 20 kDa/15 g; t¶ *p* < 0.05 o PEG 20 kDa/20 g. *n* = 3.

To analyze tissue response to ischemia, we performed RTqPCR assay on preserved pig kidneys (Figure 2). UW grafts did not show altered expression of any of the markers. Similarly, PEG 20 kDa preserved kidneys showed no significant change in target mRNA expressions. PEG 35 kDa kidneys consistently lost expression for vascular endothelium growth factor (VEGF) and VEGF receptor (Flk) at both time points (Figure 2A,C). Hypoxia inducible factor 1 alpha (HIF) expression decreased in PEG 35 kDa/30 g after 24 h. There was a trend towards early expression of Hsps 27, 70, and 90 for PEG 35 kDa/15 and 30 g/L (Figure 2B), while this expression was seen only at 24 h for PEG 35 kDa/5 g (Figure 2D). 

**Figure 3:**
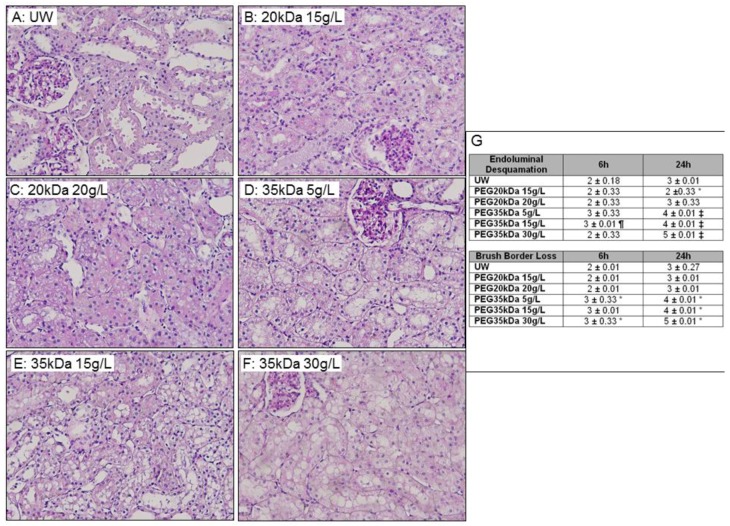
Histological Evaluation of Preserved Kidneys. (**A**–**F**) Representative Histology of preserved kidneys after 24 h preservation. Kidneys were preserved in the indicated solution for 24 h before processing for histological evaluation. Magnification: 200×; (**G**) Evaluation and scoring of histological lesions development during kidney preservation. Histological scoring was done according to the following grades: 0—no abnormality; 1—less than 10%; 2—10–25%; 3—26–50%; 4—51–75%; 5—more than 75%. Statistics: * *p* < 0.05 vs. UW, ‡ *p* < 0.05 vs. PEG 20 kDa, ¶ *p* < 0.05 vs. UW et PEG 20 kDa. *n* = 3.

Histological evaluation of preserved kidneys (Figure 3) revealed a progressive endoluminal desquamation and loss of brush border in the UW group and a similar evolution of tissue damage in PEG 20 kDa/20 g. The PEG 20 kDa/15 g group showed significant protection against endoluminal cell detachment at 24 h (*p* < 0.05 to UW). PEG 35 kDa preserved kidneys showed a higher level of damage compared to UW at both time points, with the highest level recorded for PEG 35 kDa/30 g/L (*p* < 0.05 to UW).

### 2.3. Graft Function Recovery

**Figure 4:**
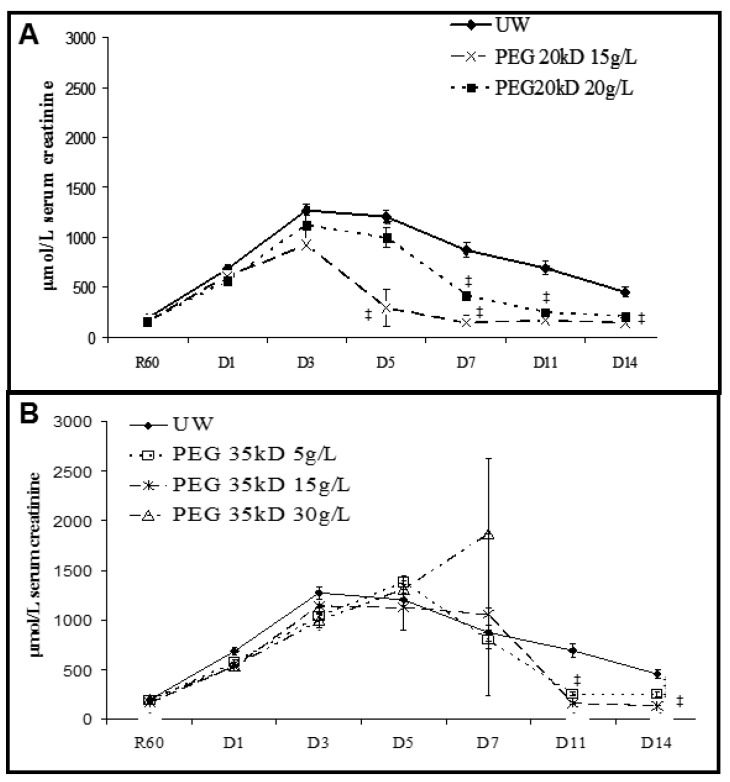
Function Recovery post-Reperfusion. Serum creatinine was measured in pigs transplanted with kidneys preserved in UW or PEG-containing solutions. (**A**) Groups UW, PEG 20kDa/15 g and PEG 20 kDa/20 g; (**B**) Groups UW, PEG 35 kDa/5 g, PEG 35 kDa/15 g and PEG 35 kDa/30 g. Shown are mean + SEM. Statistics: ‡ *p* < 0.05 to UW. *n* = 3.

We measured function recovery after transplantation (Figure 4). Compared to UW kidneys, PEG 20 kDa/20 g animals showed faster recovery (significant at day 7) reaching R60 creatinine levels by day 14 (*p* < 0.05 to UW). PEG 20 kDa/15 g demonstrated the earliest recovery, starting at day 5 and attaining R60 levels by day 7 (*p* < 0.05 to UW). In the PEG 35 kDa/15 g group, one animal was lost to primary non function, the remaining animals in PEG 35 kDa/5 g and 15 g groups showed similar function recovery compared to UW, with R60 levels recovered by Day 11 (*p* < 0.05 to UW). PEG 35 kDa/30 g showed poor performance with two primary non functions at day 7.

### 2.4. Chronic Outcome

**Figure 5:**
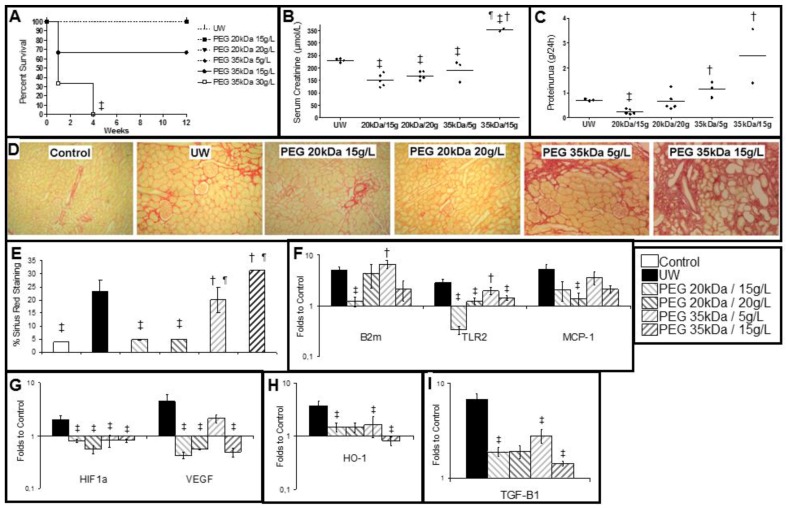
Long Term Outcome. (**A**) Kaplan-Meier curve of animal survival over a period of 3 months; (**B**) Serum creatinine (µmol/L); (**C**) Proteinuria (g/24 h); (**D**) Representative images of Sirius Red staining on tissue sections from the different groups (Magnification: 100×); (**E**): graphical representation of the amount of fibrosis as % of Red Sirius Staining; (**F**–**I**) RTqPCR on kidneys at three months. Results are expressed as folds to level of expression in Controls. Shown are mean + SEM. Statistics: ‡ *p* < 0.05 to UW, ¶ *p* < 0.05 to PEG 20 kDa/20 g/L and † *p* < 0.05 to PEG 20 kDa/15g/L. *n* = 3.

Animal survival at three months (Figure 5A) demonstrated full survival for animals with UW and PEG 20 kDa preserved kidneys, and no more animals were lost in the PEG 35 kDa/15 g group; however all animals in the PEG 35 kDa/30 g were lost by week 4 (*p* < 0.05 to other groups).

Kidney function, measured by serum creatinine (Figure 5B) and proteinuria (Figure 5C) showed decreased performance in the UW group compared to PEG 20 kDa preserved kidneys. PEG 20 kDa/15 g demonstrated the best function on both parameters (*p* < 0.05 to UW). PEG 35 kDa/5 g preservation showed lower creatinine levels than UW, however PEG 35 kDa/15 g proved deleterious in the long term, with increased serum creatinine and proteinuria (*p* < 0.05 to UW).

### 2.5. PEG 20 kDa Reduced Chronic Tubular Atrophy and Interstitial Fibrosis

We measured interstitial fibrosis and tubular atrophy (IFTA) development at 3 months by Sirius Red staining (Figure 5D,E). UW kidneys as well as PEG 35 kDa grafts showed important fibrosis (up to 20% of observed area), while PEG 20 kDa groups demonstrated significantly lower levels, comparable to controls (*p* < 0.05 to UW). 

### 2.6. PEG 20 kDa Grafts Show Decreased Activation of Lesional Pathways

Using RTqPCR, we showed that expression of class I major histocompatibility complex (MHC) molecule β-2Microglobulin (B2m) was increased in all groups except PEG 20 kDa/15 g (*p* < 0.05 to UW). Toll-like receptor 2 (TLR2) and Monocyte Chemoattractant Protein 1 (MCP1), markers of innate immunity, were increased in UW graft, while PEG grafts showed reduced expression, particularly in PEG 20 kDa kidneys (Figure 5F). Both HIF1α and VEGF were increased in UW kidneys, whereas such increase was absent in PEG grafts, with the exception of PEG 35 kDa/5 g for VEGF (Figure 5G). Oxidative stress marker Heme Oxygenase 1 (HO-1) was increased in UW grafts but not PEG-preserved kidneys (Figure 5H). Fibrosis marker Transforming growth factor β 1 (TGFβ) showed a similar pattern (Figure 5I).

## 3. Discussion

Herein we demonstrate through the use of both in vitro (hypoxia) and in vivo (ischemia reperfusion) models that organ preservation using PEG 20 kDa/15 g/L provides superior cellular and organ protection against IRI compared to UW and other PEG solutions. This is, to our knowledge, the first study comparing PEGs of different lengths and dosage in otherwise identical conditions, providing a clear evaluation of their potential. 

In vitro, cells preserved with PEGs show higher resistance to hypoxic stresses, with higher ATP content and Mitochondrial Succinate Deshydrogenase activity, coupled to lower necrosis (LDH release). Interestingly, there was a lack of effect of PEG 20 kDa/15 g in vitro at 6 h. While it is possible that the protective effect is delayed compared to the other groups, this formulation is the most performant in vivo, hence there might be a need for some injury early during IR, for instance to activate regenerative pathways. Such hypothesis will have to be further investigated in dedicated models. We further explored the intracellular effect of our conditions with RTqPCR. Markers were: (a) EGR1, found in kidneys undergoing IR in a rapid paced expression/suppression cycle in response to inflammatory cytokines [28,29], inducing a wide range of pro-rejection molecules [30]. (b) HIF1α, a major factor in the tissue adaptation to hypoxia [31,32]. (c) Von Willebrand Factor (vWF), secreted by endothelial cells in response to platelet fixation or stress, playing a key role in coagulation, a key mechanism in IRI [26,33]. (d) P47Phox, a subunit of NADPH oxidase, its expression linked to oxidative stress level [34]. (e) HSPs, protective against stresses, including apoptosis [35] and ischemia reperfusion [36], but also having a role in inflammation, as chaperones to CMH proteins [37] and potential danger signals [38]. Herein, the dichotomy in the timing of expression of these markers between UW and PEGs could highlight their two edged nature: (a) on the one hand, early expression seen in PEG-preserved cells appears protective, the cells having reserves to respond to the stresses and build the appropriate response; (b) on the other hand, delayed expression in UW would likely lay ground to a heightened amount of damage at reperfusion considering the state of the cell at this point in regards to ATP content and general survival. 

Similar RTqPCR analysis in preserved kidneys showed very different results; however the small proportion of endothelial cells in a typical cortex could explain this discrepancy. In this setting, and in regards to outcome for these grafts, stasis of expression as seen in UW preserved kidney, also observed in PEG 20 kDa groups for most markers, appears beneficial in regards to short and chronic outcome. Hsp90 expression during preservation appears to be beneficial on the long run, as PEG 20 kDa/15 g showed the best results. On the other hand, HSP expression was also present in PEG 35 kDa groups, which showed similar or worse outcomes compared to UW, hence refining of these results appears necessary. Timing of expression discriminates between doses of PEG 35 kDa: both PEG 35 kDa/15 and 30 g had early expression of HSPs, but this was delayed in PEG 35 kDa/5 g/L, the group with the best outcome. There is thus a possible association between heat shock proteins expression during preservation and outcome. Lastly, PEG 35 kDa preserved kidney showed loss of expression for HIF as well as its downstream targets VEGF and Flk, underlining an unbalanced response to hypoxia.

One limit of our in vitro investigation is the model, as we focused on endothelial cells. Endothelial cells are the first target of ischemia reperfusion lesions, and are a major cell type to protect during this injury due to its role at the interface between the organ and the recipient’s blood and immune system. It is also a key player in the revascularization of the organ, a major element of recovery. While tubular cells exhibit lesions post-IR, investigating protection of preservation solution may not be as relevant, as no true measurement of what constituents passes the glomerular has been done. Hence, we believed that endothelial cells were more appropriate to study our particular hypothesis.

Histological analysis showed that PEG 20 kDa/15 g offered some level of protection against prolonged storage lesions compared to UW, however PEG 35 kDa groups showed the most significant findings, with consistent worsening of tissue damage compared to the other groups. While both ionic composition as well as colloid can explain the differences between UW and PEG 20 kDa, only chain length differs from PEG 20 kDa and PEG 35 kDa groups. A study on the relation between chain length and level of protection [15] showed benefits from longer chains in regards to exclusion volume generation, however it did not test PEGs longer than 20 kDa. PEG binding affects the cell surface potential gradient [15] and the water structure above the cell [13], thus the structures created by PEG 35 kDa chains could be an additional stress. 

Another difference between the PEG solutions is their viscosity (Table 1), indeed both PEG 35 kDa 15 and 30 g/L, which fared the worst both ex vivo and in vivo, present a high viscosity compared to the other three, suggesting that high viscosity PEG solutions may not be suitable for proper wash-out of the organ. Viscosity is unlikely to be the only explanation however, since UW also presents a high degree of viscosity and performed better than PEG 35 kDa 15 and 30 g/L, highlighting the fact that comparison between solutions needs to include all components, and thus that our approach to determine which PEG is most appropriate for organ preservation, through the use of an otherwise identical composition, is likely to offer the best basis for comparison. Another element to take into account is the structure adopted by PEG molecules at the cell surface, indeed depending on the chain length and concentration, PEGs will adopt either a “mushroom” or a “brush” shape [39,40], which alters the osmotic balance and possibilities of interactions with extracellular elements. Herein we did not discriminate chain lengths in vitro, suggesting that the deleterious effects of PEG 35 kDa are dependent on tissue structure and the organization of endothelial cells. Additional studies are needed to determine the validity of these hypotheses. 

These results highlight the rift between in vitro and in vivo studies, and the care that should be taken when drawing conclusions from these models. Similarly, our present conclusions are at odds with previous findings from our team, comparing the performance of different PEG lengths on cellular protection against cold storage-induced injury [41]. Indeed, we reported the superiority of PEG 35 kDa over 20 kDa in vitro in terms of cell survival, resistance to oxidative stress and survival pathway activation. However, this model used a tubular epithelium cell line cold stored in normoxic atmosphere, while in the present study we present results from primary endothelial cells cold stored under a hypoxic atmosphere. There is thus a critical role played by the model. Careful choice of in vivo models is also required [24], as demonstrated herein when the deleterious effects of high doses of PEG 35 kDa were only uncovered in whole kidney models, both ex vivo and in vivo.

We confirmed the superiority of PEG 20 kDa, particularly 15 g/L, by measuring acute function recovery and chronic injury development through evaluation of IFTA and kidney function. This is likely due to the higher ability to maintain tissue structure and cellular energy content, probably allowing for faster recovery from stress. Here also, effects of chain length is evidenced, and there appear to be a dose effect with 5 g/L PEG 35 kDa offering a similar phenotype than UW, and a worsening of the outcome with the increase in PEG 35 kDa content. 

Chronic outcome is strongly correlated with immune response [42] a process impaired by the properties of PEGs [43]. RTqPCR evaluation of immune markers indicates a level of protection from PEGs, particularly 20 kDa chains. PEG 35 kDa-preserved kidneys show trend towards lowered expression of immune markers, suggesting some protection from PEG 35 kDa/5 g over UW, however the other PEG 35 kDa groups provided inadequate protection. These suggest a dependence of immune camouflage on PEG chain length. A recent report questioned the PEGs’ immunocamouflage potential, through in vitro experiments involving peripheral blood monocyte cells incubated with PEGs or different length and dosage [44]. Only anchored PEGs could provide protection against antigen recognition, however incubation times with free PEGs were short (1 h), and thus possibly not sufficient for adsorption of the PEG. There is thus a need to confirm these findings with a more appropriate model.

HIF-1α has been described to enhance fibrogenesis as well as epithelial to mesenchymal transition (EMT) [32], particularly during chronic stress. Herein, expressions of HIF1α and VEGF are consistent with the advanced lesions in UW grafts, and their absence in PEG 20 kDa-groups is in accordance to reduced fibrosis. Expression in PEG 35 kDa kidneys does not follow this pattern, similarly found for TGFβ, a potent inducer of EMT and fibrosis [45]. These results confirm the slight superiority of PEG 35 kDa/5 g over UW. However, absence of expression in highly fibrotic kidneys such as PEG 35 kDa/15 g-preserved grafts is counter-intuitive and highlight the limitations of RTqPCR as a standalone diagnostic tool [46,47]. 

HO-1 is protective against ischemia reperfusion injury [48], and chronic expression appears to lower fibrosis development [49]. Absence of overexpression in PEG 20 kDa kidneys can be attributed to the absence of chronic lesion, while in UW grafts HO1 expression could indicate repair mechanisms in place to slow the development of fibrosis. In this light, absence of expression in PEG 35 kDa/15 g kidneys suggests a more advanced lesion, having exhausted repair attempts. HO1 could thus be an interesting marker to discriminate kidneys in advanced state of lesion.

Our in vivo study appears limited as we did not investigate the optimization of UW with PEG instead of HES. However, previous work [20,50,51] demonstrated the superiority of PEG-based solutions such as SCOT^®^ and IGL. Moreover, these studies also showed that solutions mimicking the ionic composition of the extracellular milieu were more performant for organ protection. Furthermore, our team experimented with intracellular type solutions (similar to UW) using PEG instead of HES [52]. While performance with PEG was better, we obtained results that were inferior to extracellular solutions. We thus decided to measure the possible optimization of these new, fourth generation solutions, rather than attempt to change a solution with an older formulation.

## 4. Materials and Methods

### 4.1. In Vitro Experiments

Primary porcine kidney endothelial cells were used as previously described [33], hypoxia was achieved by incubation in hypoxic atmosphere (Bactal 2 gaz, 0% O_2_, 5% CO_2_, and 95% N_2_, Air Liquide, Paris, France) at 4 °C in UW or PEG solutions. Controls cells were cultured in regular media in normoxic atmosphere (20% O_2_) for equivalent lengths of time. Assays were: (i) necrosis: ratio supernatant LDH/intracellular LDH (tested on automated analyzer, Modular analytics P, Roche); (ii) Mitochondrial Succinate Deshydrogenase activity: XTT kit (Roche, Meylan, France); (iii) Intracellular ATP: Intracellular ATP: ATPlite 1step Luminescence Assay kit (Perkin-Elmer, Villebon-sur-Yvette, France); following the manufacturer’s guidelines. Reactions were quantified by spectrophotometer (Victor3, Perkin-Elmer, Villebon-sur-Yvette, France).

### 4.2. In Vivo Surgical Procedures and Experimental Groups

Large white male pigs (INRA/GEPA, Surgères, France) were prepared as previously described [53] in accordance with French guidelines of the Ethical Committee for Human and Animal Studies (comity number C2EA-84, Protocol number CE2012-4, accepted the 20 December 2012).

The kidney was collected, cold flushed, and preserved for 24 h before transplantation, when the left kidney was nephrectomized to mimic nephron mass in transplanted situation. Surgical teams were blinded to protocols. Time for anastomosis was 30 ± 5 min, blood loss was minimal and no post-operative complications were observed. Six groups were studied: 1-UW: UW solution (low-Na^+^/high-K^+^) with 50 g/L HES; 2-PEG 20 kDa/15 g: SCOT^®^ solution (plasma-like high-Na^+^/low-K^+^) with 15 g/L PEG 20 kDa; 3-PEG 20 kDa/20 g, 4-PEG 35 kDa/5 g; 5-PEG 35 kDa/15 g; 6-PEG 35 kDa/30 g. Controls were sham-operated animals. Due to the number of conditions tested, and the extensive in vitro testing which already permitted to evaluate differences between groups, we limited the study to *n* = 3 at each time point in order to respect the 3R rule. An ex vivo series was performed in which serial wedge biopsies were collected on cold preserved kidneys at 6 and 20 h cold static preservation.

### 4.3. Function and Histopathology

Serum creatinine and urinary proteins were measured as previously described [3]. Corticomedular samples were collected, frozen or fixed in 10% formalin and embedded in paraffin. All sections were examined under blind conditions by a pathologist. Brush border loss and endoluminal detachment were assessed using a semi-quantitative 6-point scale: 0—no abnormality; 1—mild lesions affecting less than 25% of kidney samples; 2—lesions affecting 25–50% of kidney samples; 3—lesions affecting 51–75% of kidney samples; 4—lesions affecting more than 75% of kidney samples and 5—extensive necrosis and renal damage [54].

A standard procedure was used to estimate the level of tubulointerstitial fibrosis using Picro-Sirius staining [55].

### 4.4. Real Time PCR

We used Trizol for RNA extraction (Fisher Scientific, Illkirch-Graffenstaden, France). Genomic DNA was removed using DNA-free kit (Applied Biosystems, Foster City, CA, USA) and first-strand reverse transcription (Applied) was performed. Real Time PCR assays were performed on an ABI Prism 7300 (Applied). Porcine primers (Table S2) were designed using OligoPerfect™ (Invitrogen, Carlsbad, CA, USA). Expression level was obtained with the 2(−ΔΔ*C*_t_) Method.

### 4.5. Statistical Methods

Results are shown as mean ± SEM. For the statistical analysis among groups, we used NCSS software (NCSS LLC, Kaysville, UT, USA) and one-way ANOVA analysis with the Tukey-Kramer test for multiple comparisons in case of normality (Skewness, Kurtosis and Omnibus tests), and equality of variance (Modified-Levene Equal-Variance Test) and Kruskal-Wallis Multiple-Comparison *Z*-Value Test (Dunn’s Test) in case these parameters were not met. Statistical significance was accepted for *p* < 0.05.

## 5. Conclusions

The present study demonstrates superiority of PEG 20 kDa/15 g preservation over UW. Moreover, our results highlight the importance of PEG length and dosage for optimal protection against ischemia reperfusion, as PEG 20 kDa/20 g showed inferior performances compared to 15 g/L and only low dosage of PEG 35 kDa (5 g) offered some degree of protection compared to UW, with worsening of outcomes in higher doses. As all other parameters were equal in the PEG groups, we can conclude that chain length is a critical factor in PEG performance, and that longer chains such as PEG 35 kDa must be used at low doses, such as found in the IGL1 solution (1 g/L). Hence, this novel compound can be a precious ally in optimizing graft preservation; however, correct assessment of optimal length and concentration is critical.

## Figures and Tables

**Table 1:** Physical properties of the different preservation solutions.

Solution	PEG Concentration (mM)	Density (g/cm^3^)	Viscosity (Cst)
UW	0	1.047	3.22
PEG 20kDa/15 g	0.75	1.010	1.41
PEG 20 kDa/20 g	1	1.011	1.55
PEG 35kDa/5 g	0.14	1.008	1.23
PEG 35 kDa/15 g	0.43	1.010	1.90
PEG 35 kDa/30 g	0.85	1.032	4.56

## Supplementary Materials

Supplementary materials can be found at http://www.mdpi.com/1422-0067/19/2/454/s1.

## Abbreviations

EGR1	Early growth response factor 1
Flk	VEGF receptor
HES	Hydroxyethyl starch
HIF1α	Hypoxia inducible factor 1 alpha
IGL1	Institut George Lopez
LDH	Lactate DesHydrogenase
MHC	major histocompatibility complex
p47	NADPH oxidase subunit Phoxp47
PEG	Polyethylene glycol
SCOT	Solution de Conservation des Organes et des Tissus
TGFβ	Transforming growth factor β 1
UW	University of Wisconsin
VEGF	Vascular endothelium growth factor
vWF	Von Willebrand factor
XTT	2,3-Bis-(2-Methoxy-4-Nitro-5-Sulfophenyl)-2*H*-Tetrazolium-5-Carboxanilide
