# Supplementary material for: The Optimal PEG for Kidney Preservation: A Preclinical Porcine Study
Source: Int J Mol Sci. 2018 Feb 3;19(2):454. doi: 10.3390/ijms19020454 (PMC5855676; doi:10.3390/ijms19020454)
Supplement: Supplementary file 1 [file ijms-19-00454-s001.pdf]

Table S1: Composition of the preservation solutions.

Compounds	Blood	UW	PEGs
Ions/Molecules (mM)			
Na^+	140	30	118
K^+	5	125	5
Mg^{2+}	0.8	5	1.20
Ca^{2+}	2.5		1.75
Cl^-	104		
Fe(NO3)3, 9 H_2O			
$SO4^{2-}$	1.4	5	
H_2PO4^{2-}	3.2	25	
HCO3-	25		25
HEPES			
Glucose	7		11
Raffinose		30	
Ribose			
lactobionate		100	
adenosine		5	
glutathion		4	
allopurinol		1	
Histidine			
Mannitol			
Glutamate			
Pyruvate de Na			
Tryptophan			
α-Ketoglutarate			
Glutamine			
Vitamins			
Amino acids			
Colloïdes (g/L)			
HES		50	
PEG 20 kDa			Varies
PEG 35 kDa			varies
Albumine	42		
Globuline	24		
Physico-chimie			
pH	7.4	7.3	7.3
Viscosité (cSt)	1.6	2.4	1.6
Osmolarité (mOsm)	308	320	320

Supplementary Table S2: Primer sequences for RT-PCR analysis.

Gene	Forward	Reverse
18S	AGCCTGCGGCTTAATTTGAC	AACCAGACAAATCGCTCCAC
β2microglobulin	TCCGCCCCAGATTGAAATT	TGCTCCGCGTTCATCTTCT
EGR1	GGGCACCCCAGACCA	CGACTGCGTGGCAAAG
Flk-1	GGACCATTTCAAACCTCAATGTG	ACCATCAGGAACAAACCTCTTTTC
Hif-1 α	TGGCAGCAATGACACAGAAAC	GAGGCAGGCAATGGAGACAT
HSP27	CGAGGAGCTGACGGTCAAG	GCAGCGTGTATTTTCGAGTGAA
HSP70	TTTTCTGCCTCCACAAACG	ATCTAATGCAAAGAATACAGTCCAG
HSP90	CCGGTGCCGATATCTCGAT	GCGACCAGGTACGCAGAGTAG
HO-1	GCTGAGAATGCCGAGTTCATG	GGACGCCATCACCAGCTTAA
MCP1	TCTCCAGTCACCTGCTGCTAT	TGCTTCTTTAGGACACTTGCTG
P47	CATCATCCTGCAGACGTACC	TCGTTCTTCTCCACGACATC
TGFβ	CGAGCCAGAGGCGGACTA	TATCATAGATTTGGTTGCCGCTTT
TLR2	GGGCTCTGTGCCACCACTT	TGGAGCCAGGCCCACAT
VEGF	GCCCACTGAGGAGTTCAACATC	GGCCTTGGTGAGGTTTGATC
vWF	GCAGCAAGAGGAGTCTGAGC	GGCAGGTCTTGGCACACT
